# Acrylamide and Its Metabolite Glycidamide Induce Reproductive Toxicity During In Vitro Maturation of Bovine Oocytes

**DOI:** 10.3390/toxics13030223

**Published:** 2025-03-19

**Authors:** Marwa El-Sheikh, Ahmed Atef Mesalam, Ayman Mesalam, Il-Keun Kong

**Affiliations:** 1 Department of Microbial Biotechnology, Biotechnology Research Institute, National Research Centre (NRC), Dokki, Cairo 12622, Egypt; 2 Department of Therapeutic Chemistry, Pharmaceutical and Drug Industries Research Institute, National Research Centre (NRC), Dokki, Cairo 12622, Egypt; ahmedatefmesalam@hotmail.com; 3 Department of Theriogenology, Faculty of Veterinary Medicine, Zagazig University, Zagazig 44519, Egypt; aymanmesalam@gmail.com; 4 Division of Applied Life Science (BK21 Four), Gyeongsang National University, Jinju 52828, Republic of Korea; 5 Institute of Agriculture and Life Science, Gyeongsang National University, Jinju 52828, Republic of Korea; 6 The King Kong Corp., Ltd., Gyeongsang National University, Jinju 52828, Republic of Korea

**Keywords:** acrylamide, glycidamide, oocyte, autophagy, apoptosis, epigenetics

## Abstract

Acrylamide (ACR) and its metabolite glycidamide (GLY) are contaminants with known toxic effects, especially in reproductive systems. However, the mechanisms underlying their embryotoxic effects remain inadequately understood. In the current study, we investigated the effects of ACR and GLY exposure on oocyte and embryo developmental competence, focusing on DNA damage, apoptosis, autophagy, and epigenetic regulation. Oocytes were exposed to varying concentrations of ACR and GLY during in vitro maturation. The results demonstrated that both ACR and GLY significantly reduced cleavage and blastocyst developmental rates in a dose-dependent manner. Consequently, treated oocytes exhibited actin organization disruption, increased DNA damage, and heightened apoptosis compared to the control. Autophagy-related markers, including LC3A, LC3B, and ATG7, were significantly elevated in the treatment groups. Moreover, both ACR and GLY compounds altered the expression of the epigenetic and MAPK signaling pathway regulators, such as DPPA3, EZH1, EZH2, EED, DUSP1, and ASK1. These disruptions collectively impaired embryonic development. This study underscores the adverse effects of ACR and GLY on reproductive health, driven by oxidative stress, genotoxicity, dysregulated autophagy, and epigenetic alterations.

## 1. Introduction

Acrylamide (ACR) is a water-soluble compound commonly used in various industries such as paper manufacturing, wastewater treatment, and cosmetics production [1,2]. It is also formed during high-temperature cooking of starchy foods, including potato fries, roasted barley tea, black olives, coffee, and nuts, making it a widespread contaminant [3,4,5]. Additionally, significant amounts of ACR are present in tobacco smoke, with smokers experiencing over 50% higher ACR exposure compared to non-smokers [6]. ACR is considered cytotoxic, neurotoxic, and genotoxic, with a high likelihood of being carcinogenic [7]. Given its ubiquity in food and tobacco, exposure to ACR is almost inevitable, raising substantial public health concerns about the long-term exposure effects [8].

Upon ingestion, ACR is metabolized by cytochrome P450 into glycidamide (GLY), a more reactive and toxic epoxide metabolite [9]. GLY readily forms DNA adducts, which are associated with mutagenic and carcinogenic effects in various tissues, including reproductive organs [10,11,12]. These processes induce oxidative stress, increase apoptosis, and cause DNA damage, severely compromising reproductive health [13,14,15]. Both ACR and GLY accumulate in tissues and disrupt biological functions [4,14]. Scientific findings suggest that ACR and GLY exposure increases the mutations rates in sperm cells, with GLY having a more pronounced impact on sperm viability [16,17]. In murine models, prolonged exposure to ACR has been linked to increased sperm abnormalities, reduced fertility, and lower litter sizes [18,19]. Also, male rats exposed to ACR exhibited diminished sexual behavior, reduced sperm counts, testicular atrophy, and degeneration of the seminiferous epithelium [20,21]. However, the impact of ACR and GLY on oocyte and embryo quality remains insufficiently understood.

Oocyte quality is assessed based on its ability to mature, achieve fertilization, and produce viable offspring [22]. Proper meiotic division and chromosomal integrity are essential for oocyte maturation, and disruptions in these processes can severely impair fertility [23]. Studies have shown that these compounds, ACR and GLY, interfere with essential cellular processes, such as spindle formation and chromosomal segregation during meiosis, which are crucial for successful oocyte maturation and embryogenesis [14,15]. ACR has been shown to disrupt critical cytoskeletal components, including intermediate filaments, impairing oocyte maturation by inducing apoptosis in mice [4,24]. Oral ACR intake has been associated with reduced ovarian weight and fewer germinal vesicle-stage oocytes in mice [15]. Additionally, ACR exposure negatively affected granulosa cell proliferation, decreased corpora lutea numbers, and reduced progesterone production, further compromising the hormonal environment required for optimal oocyte development and fertility [25]. Prolonged exposure to ACR and GLY has also been linked to lower fertility rates and diminished embryo viability in experimental models [14]. The bovine oocyte serves as a well-established model for studying mammalian reproductive toxicology due to its structural and physiological similarities to human oocytes, making it highly relevant for translational research [26,27]. Additionally, bovine embryos share key developmental characteristics with human embryos, providing valuable insights into early embryogenesis and toxicological impacts [26,28]. Moreover, assessing the toxic effects of environmental contaminants on bovine oocytes and embryos is crucial for evaluating reproductive hazards in the livestock industry, as exposure to such substances can compromise fertility and productivity [29,30]. 

Epigenetic modifications play a crucial role in regulating gene expression during oocyte maturation and early embryonic development. DNA methylation and posttranslational histone modifications are key mechanisms that establish the epigenetic landscape required for proper oocyte and embryo development [31]. DNA methylation orchestrates gene expression by making specific genes for activation or repression, influencing critical processes such as cleavage, differentiation, and preimplantation development [32,33]. ACR exposure has been shown to reduce DNA methylation levels in mouse oocytes, impairing this regulatory mechanism [15]. Furthermore, histone modifications, such as H3K9, H3K4, and H3K27, are critical for chromatin organization and transcriptional activity [34]. ACR treatment disrupted these modifications, leading to defects in chromosome condensation and segregation during meiosis, ultimately impairing oocyte quality and delayed maturation [32,35].

While the effects of both ACR and GLY on male reproductive health are documented, their impact on female reproductive health remains underexplored. In this study we evaluate the effects of ACR and its metabolite GLY on bovine oocyte and embryo development in vitro, with a specific focus on DNA damage, autophagy, and epigenetic alterations.

## 2. Materials and Methods

### 2.1. Experimental Design and Reagents

The experimental design of this study included three phases. Initially, the effect of ACR on bovine embryo development was assessed by adding it to the in vitro maturation (IVM) medium at various concentrations (0, 250, 500, and 1000 µM). Embryo cleavage rates on day 4 and blastocyst formation on day 8 post-fertilization were recorded. The second phase investigated the effect of GLY, a main metabolite of acrylamide, on embryo development at concentrations of 0, 1, 10, 100, and 1000 µM. In the third phase, the study encompassed three groups corresponding to ACR treatment (500 µM), GLY treatment (10 µM), and an untreated control group. In each experiment, oocytes were plated at approximately 50 oocytes per well, with four replicates per treatment group. All experiments involving bovine oocytes and embryos were carried out with the approval of the Animal Care and Use Committee of GNU (GNU-230425-A0088). All chemicals and reagents used in this study were obtained from Sigma-Aldrich (St. Louis, MO, USA), unless specified.

### 2.2. Oocyte Collection and In Vitro Maturation (IVM)

Bovine ovaries were collected from a local abattoir and transported to the laboratory in saline solution within two hours post-collection. Cumulus-oocyte complexes (COCs) were retrieved from antral follicles using an 18-gauge needle attached to a vacuum pump. COCs with at least three layers of cumulus cells were collected and washed three times in TL-HEPES medium and then transferred to in vitro maturation (IVM) medium. The COCs were then cultured for 24 h at 38.5 °C in a humidified atmosphere containing 5% CO_2_.

### 2.3. In Vitro Fertilization (IVF) and Culture

Bovine semen was washed in D-PBS and re-suspended in TL-FERT medium (which consisted of Tyrode lactate solution supplemented with 6 mg/mL bovine serum albumin (BSA), 22 µg/mL sodium pyruvate, 95 µg/mL L-Cysteine, 100 IU/mL penicillin, and 0.1 mg/mL streptomycin) containing 20 µg/mL heparin to achieve a final concentration of 1 × 10^6^ sperm/mL. After IVM, the COCs and sperm were co-incubated for 18 h at 38.5 °C and 5% CO_2_. Following IVF, presumptive zygotes were denuded of cumulus cells by repeated pipetting and cultured in culture medium [36] at 38.5 °C and 5% CO_2_.

### 2.4. Visualization of Cytoskeleton

Oocytes were denuded across four separate replicates (*n* = 10 per group) and fixed in 4% paraformaldehyde followed by incubation for two hours in blocking buffer containing 10% donkey serum and 3% bovine serum albumin (BSA) in phosphate-buffered saline (PBS). Oocytes were stained with Alexa Fluor 488-phalloidin for three hours then washed in PBS, mounted on glass slides, and visualized using a confocal laser-scanning microscope (Olympus, Tokyo, Japan), while optical densities of the cytoskeletal structures were quantified using ImageJ software version 1.54 (https://imagej.nih.gov/ij).

### 2.5. Terminal Deoxynucleotidyl Transferase dUTP Nick End Labeling (TUNEL)

The TUNEL assay was conducted using the In Situ Cell Death Detection Kit (Roche Diagnostics Corp., Indianapolis, IN, USA), following the manufacturer’s protocol. Day-8 embryos (*n* = 15 per group) were washed twice in 0.3% polyvinylpyrrolidone in 1 M PBS (PVP-PBS) and permeabilized with 0.5% Triton X-100 at room temperature. After 30 min, embryos were washed twice in PVP-PBS and incubated with fluorescently conjugated terminal deoxynucleotide transferase dUTP for 1 h at 37 °C. The nuclei were stained with 10 µg/mL Hoechst 33342 for 10 min and then the embryos were washed in PVP-PBS and mounted on glass slides. TUNEL-positive cells, indicating apoptosis, were visualized as red signals under an epifluorescence microscope (Olympus IX71, Tokyo, Japan).

### 2.6. Immunofluorescence Analysis

Oocytes and day-8 blastocysts (*n* = around 15 per group) were fixed in 4% paraformaldehyde for 15 min at room temperature. Samples were rinsed three times with 0.3% PVP-PBS and permeabilized with 0.5% Triton X-100 for 20 min at room temperature. Oocytes and embryos were washed three times with PVP-PBS and incubated in blocking buffer consisting of 10% donkey serum and 3% BSA in PBS for 4 h at room temperature. Subsequently, samples were incubated overnight at 4 °C with primary antibodies against caspase-3 (Santa Cruz Biotechnology, Santa Cruz, CA, USA) and LC3B (Abcam, Cambridge, Cambs, UK), both diluted 1:200 in a solution of 3% BSA and 0.1% Tween 20 in PBS. Samples were then washed three times with PVP-PBS and incubated for 90 min at room temperature with FITC-conjugated secondary antibodies (diluted 1:100, Santa Cruz Biotechnology). Nuclei were stained with DAPI (1:100 in D-PBS) for 10 min, followed by three washes with PVP-PBS. The stained oocytes and embryos were mounted on glass slides and analyzed using a confocal microscope (Olympus, Tokyo, Japan). Integrated optical densities were quantified using ImageJ software.

### 2.7. RNA Extraction and RT-qPCR

Total RNA was extracted from oocytes (*n* = 50; triplicate) using the Arcturus PicoPure RNA Isolation Kit (Arcturus, Foster, CA, USA) according to the manufacturer’s protocol. RNA concentrations were measured using a NANO DROP 2000c spectrophotometer (Thermo Fisher Scientific, Waltham, MA, USA). First-strand complementary DNA (cDNA) was synthesized using a reverse transcription kit (Bio-Rad Laboratories, Hercules, CA, USA), according to the manufacturer’s instructions. Quantitative real-time PCR (qPCR) was performed on real-time PCR detection system (Bio-Rad Laboratories, Hercules, CA, USA) under the following thermal cycling conditions: initial denaturation at 95 °C for 3 min, followed by 44 cycles of 95 °C for 15 s, 58 °C for 20 s, and 72 °C for 30 s. Gene expression levels were quantified using the 2^−ΔΔC(t)^ method and the results were normalized for the reference gene *GAPDH* and expressed as relative expression (n-fold differences). The target genes analyzed in this study (Table S1) included the following oocyte developmental markers: growth differentiation factor 9 (*GDF9*), bone morphogenetic protein 15 (*BMP15*), meiosis arrest female 1 (*MARF1*); the following epigenetic modifiers: DNA methyltransferase 1 alpha (*DNMT1A*), DNA methyltransferase 3 alpha (*DNMT3A*), DNA methyltransferase 3 beta (*DNMT3β*), euchromatic histone-lysine N-methyltransferase 2 (*G9A*), SET domain bifurcated histone lysine methyltransferase 1 (*SETDB1*), suppressor of variegation 3–9 homolog 2 (*SUV39H2*), developmental pluripotency-associated 3 (*DPPA3*), enhancer of zeste homolog 1 (*EZH1*), enhancer of zeste homolog 2 (*EZH2*), suppressor of zeste 12 homolog (*SUZ12*), embryonic ectoderm development (*EED*), BCL2-associated X (*BAX*); the following inflammation/apoptosis and cell cycle regulators: B-cell lymphoma 2 (*BCL2*), nuclear factor kappa B (*NFkB*), thioredoxin (*TXN*), caspase-3, caspase-9, inducible nitric oxide xynthase (*iNOS*), cytochrome C (*CYTC*), cyclooxygenase 2 (*COX2*), cyclin-dependent kinase inhibitor 1 (*P21*), cyclin-dependent kinase inhibitor 1B (*P27*); the following DNA repair and damage response mechanisms: 8-oxoguanine DNA glycosylase (*OGG1*), tumor protein P53 (*P53*), protein phosphatase 6 (*PP6*); the following autophagy-related genes: beclin-1 (*BECN1*), microtubule-associated proteins 1A/1B light chain 3A (*LC3A*), microtubule-associated proteins 1A/1B light chain 3B (*LC3B*), autophagy-related 5 (*ATG5*), autophagy-related 7 (*ATG7*), BCL2-interacting protein 3 (*BNIP3*), lysosomal-associated membrane protein 1 (*Lamp1*), lysosomal-associated membrane protein 2 (*Lamp2*); and the following signal transduction pathways: dual-specificity phosphatase 1 (*DUSP1*), zpoptosis signal-regulating kinase 1 (*ASK1*), apoptosis signal-regulating Kinase 3 (*ASK3*).

### 2.8. Statistical Analysis

Data were analyzed using GraphPad Prism software version 6 (GraphPad Software, San Diego, CA, USA). Differences between groups were evaluated using one-way ANOVA followed by Dunnett’s multiple comparison test. Data of compassing experiments included ACR and/or GLY treatment and control groups were analyzed using Student’s *t*-test. The results are presented as mean ± SEM, with statistical significance set at *p* < 0.05.

## 3. Results

### 3.1. Effect of Acrylamide and Glycidamide on Bovine Embryo Development

**Figure 1:**
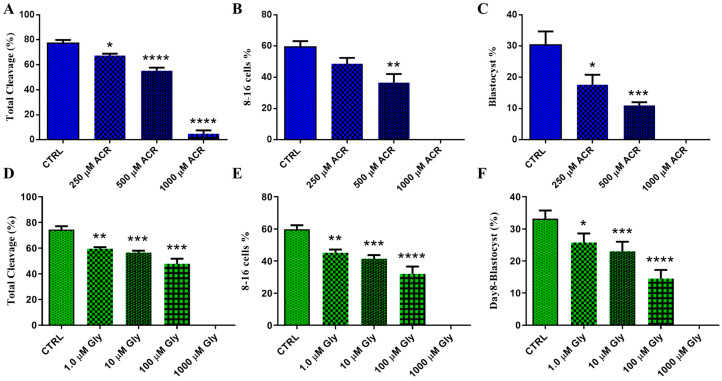
Effect of ACR and GLY on bovine embryo developmental competence. (**A**–**C**) Embryo cleavage and blastocyst development rates after exposure to varying concentrations of ACR (0, 250, 500, and 1000 µM). (**D**–**F**) Embryo cleavage and blastocyst development rates after exposure to varying concentrations of GLY (0, 1, 10, 100, and 1000 µM). The comparison between experimental groups versus the control was performed using Dunnett’s multiple comparison test. Statistical significances are presented as * (*p* < 0.05), ** (*p* < 0.01), *** (*p* < 0.001), and **** (*p* < 0.0001) and the values are shown as the mean values ± SEM.

The developmental competence of embryos derived from oocytes treated with ACR and GLY was evaluated. The ACR-treated group exhibited significantly lower cleavage and developmental rates to the blastocyst stage in a dose-dependent manner. Specifically, treatments with 250 µM and 500 µM ACR resulted in a significant reduction in total cleavage rates (67.4 ± 1.44 and 55.2 ± 2.54, respectively, vs. 77.4 ± 2.48 in the control group) and blastocyst development rates (17.6 ± 3.22 and 11 ± 1, respectively, vs. 30.4 ± 4.27 in the control group) (Figure 1A–C). Moreover, treatment with 1000 µM ACR caused complete arrest at the 2–4 cell cleavage stage, with no blastocyst development. Similarly, embryos from the GLY-treated group showed significantly lower cleavage and developmental rates to the blastocyst stage in a dose-dependent manner. The 1 µM, 10 µM, and 100 µM GLY treatments resulted in significantly reduced total cleavage rates (59.5 ± 1.26, 56.5 ± 1.55, and 47.75 ± 4.03, respectively, vs. 74.5 ± 2.66 in the control group) and blastocyst rates (25.75 ± 2.84, 23 ± 3.03, and 14.5 ± 2.72, respectively, vs. 33.25 ± 2.5 in the control group) (Figure 1D–F). Treatment with 1000 µM GLY resulted in complete arrest at the zygote stage, with no cleavage and blastocyst development.

### 3.2. Effect of Acrylamide and Glycidamide on Actin Cytoskeleton Stabilization and Oocyte Quality

**Figure 2:**
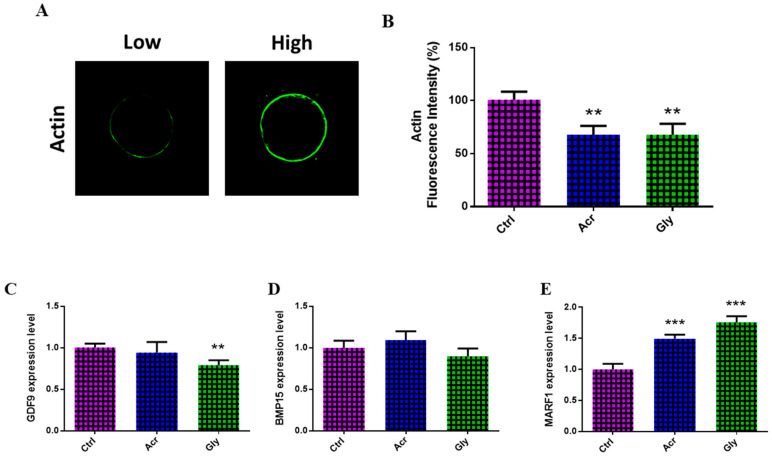
Effect of ACR and GLY at 500 µM and 10 µM dose, respectively, on the quality of bovine oocytes. (**A**) Representative images of F-actin high and low fluorescence intensity in oocytes stained with Alexa Fluor 488 phalloidin. (**B**) Fluorescence intensity analysis of the cytoplasm and zona pellucida/oolemma, showing a significant reduction in F-actin fluorescence intensity following ACR and GLY treatment. (**C**) Relative expression levels of *GDF9*, (**D**) *BMP15*, and (**E**) *MARF1* genes in oocytes treated with ACR and GLY, as determined by RT-qPCR. The comparison between ACR or GLY groups versus the control was performed using Student’s *t*-test. Statistical significances are represented as ** (*p* < 0.01), and *** (*p* < 0.001),, with values expressed as the mean ± SEM. Original magnification 100×.

Based on the above-mentioned results, three groups corresponding to ACR treatment (500 µM), GLY treatment (10 µM), and an untreated control group were selected for the following investigations. As polar body extrusion and cytokinesis critically rely on the presence of actin filaments, the actin cytoskeleton in oocytes was analyzed following treatment with ACR and GLY using phalloidin-based staining. The results indicated a disruption in actin organization in both ACR- and GLY-treated oocytes, with a significant decrease in fluorescence intensity corresponding to actin cytoskeleton (F-actin; Figure 2A,B). The expression of genes related to oocyte maturation (*GDF9*, *BMP15*, and *MARF1*) was further analyzed following ACR and GLY treatments using RT-qPCR. Oocytes exposed to ACR and GLY showed a significant upregulation in the expression of *MARF1* (Figure 2E). However, GLY-treated oocytes exhibited a significant decrease in *GDF9* expression (Figure 2C).

### 3.3. Impact of Acrylamide and Glycidamide on Key Epigenetic Regulators

**Figure 3:**
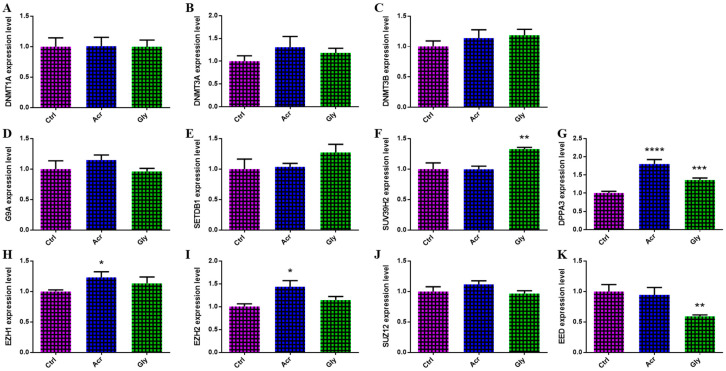
Effects of ACR and GLY on epigenetic regulator gene expression in bovine oocytes. (**A**–**K**) Relative mRNA levels of epigenetic regulation-related genes following ACR and GLY treatments. The comparison between ACR or GLY groups versus the control was performed using Student’s *t*-test. Statistical significance is presented as * (*p* < 0.05), ** (*p* < 0.01), *** (*p* < 0.001), and **** (*p* < 0.0001), with values expressed as mean ± SEM.

The expression of epigenetic regulation-related genes (*DNMT1A*, *DNMT3A*, *DNMT3β*, *G9A*, *SETDB1*, *SUV39H2*, *DPPA3*, *EZH1*, *EZH2*, *SUZ12*, and *EED*) was analyzed in oocytes following ACR and GLY treatments using RT-qPCR. Among the tested genes, the mRNA levels of *DPPA3*, *EZH1*, and *EZH2* exhibited a significant increase in oocytes treated with ACR (Figure 3). In addition, GLY-treated oocytes displayed a significant increase in the expression of *SUV39H2* and *DPPA3* and a decrease in *EED* expression (Figure 3).

### 3.4. Effect of Acrylamide and Glycidamide on Expression of Genes and Protein Involved in Apoptosis and DNA Damage Responses

**Figure 4:**
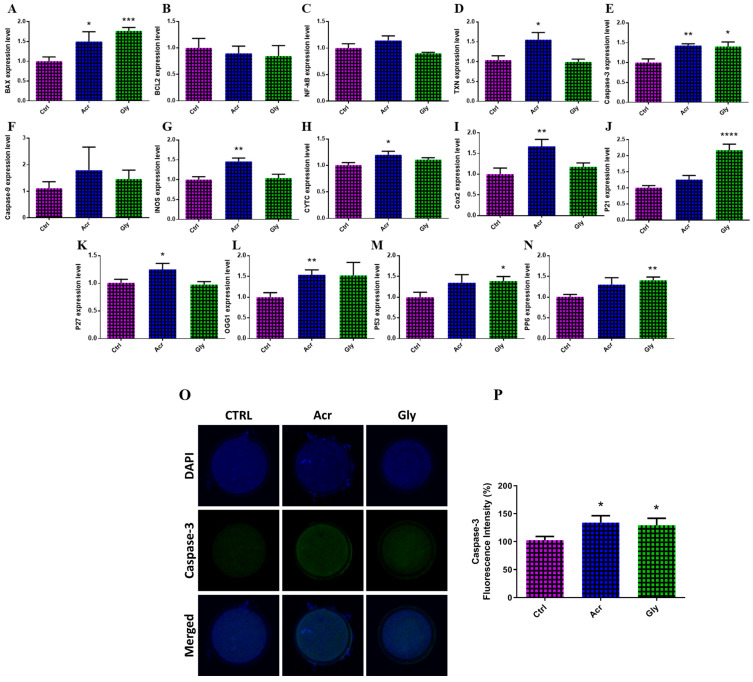
Effect of ACR and GLY on apoptosis and DNA damage in bovine oocytes. (**A**–**N**) Transcription pattern of genes associated with oxidative stress and DNA damage in oocytes. (**O**) Representative images displaying bovine oocytes stained with DAPI and caspase-3. (**P**) Fluorescence intensity following caspase-3 staining. The comparison between ACR or GLY groups versus the control was performed using Student’s *t*-test. Statistical significances are indicated as * (*p* < 0.05), ** (*p* < 0.01), *** (*p* < 0.001), and **** (*p* < 0.0001). Values are shown as mean ± SEM. Original magnification 100×.

The DNA damage in oocytes was evaluated by analyzing the transcription pattern of the following genes: *BAX*, *BCL2*, *NF-KB*, *TXN*, *caspase-3*, *caspase-9*, *iNOS*, *CYTC*, *COX2*, *P27*, *OGG1*, *P21*, *P53*, and *PP6*. As shown in Figure 4, ACR-treated oocytes showed significantly higher expression levels of DNA damage-related markers, including *BAX*, *TXN*, *caspase-3*, *iNOS*, *CYTC*, *COX2*, *P27*, and *OGG1*, compared to control oocytes. Similarly, GLY-treated oocytes exhibited increased expression of *BAX*, *caspase-3*, *P21*, *P53*, and *PP6* (Figure 4). Additionally, the levels of caspase-3, an apoptosis-related protein, were measured in oocytes exposed to ACR and GLY using immunofluorescence analysis. Oocytes exposed to ACR showed a significant increase in caspase-3 expression, suggesting higher levels of apoptosis (Figure 4O). Similarly, GLY treatment led to increased caspase-3 levels, though the increase was less pronounced compared to the ACR group (Figure 4O).

### 3.5. Effect of Acrylamide and Glycidamide on mRNA and Protein Levels of the Autophagy-Specific Markers

**Figure 5:**
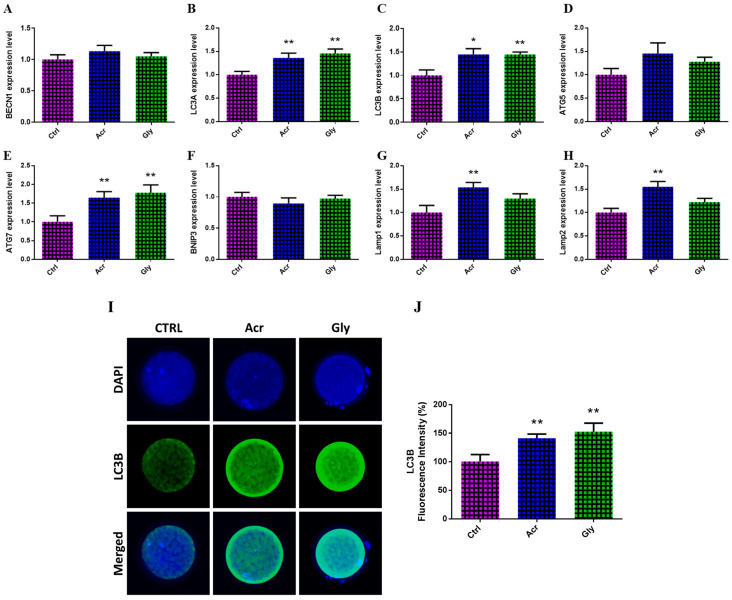
Autophagy induction in oocytes treated with ACR and GLY. (**A**–**H**) The relative expression of autophagy-related genes in oocytes treated with ACR and GLY using RT-qPCR. (**I**) Representative images displaying bovine oocytes stained with DAPI and LC3B. (**J**) LC3B protein levels measured by immunofluorescence. The comparison between ACR or GLY groups versus the control was performed using Student’s *t*-test. Statistical significance is presented as * (*p* < 0.05) and ** (*p* < 0.01), with values shown as mean ± SEM. Original magnification 100×.

The impact of ACR and GLY on autophagy in oocytes was assessed by measuring the protein levels of LC3B and the transcription pattern of autophagy-related genes. As seen in Figure 5, ACR treatment significantly upregulated the expression of *LC3A*, *LC3B*, *ATG7*, *Lamp1*, and *Lamp2* genes. Similarly, GLY treatment showed a significant increase in the expression of *LC3A*, *LC3B*, and *ATG7* genes (Figure 5A–H). At the protein level, both ACR and GLY treatments led to an increase in LC3B protein content, with GLY having a more pronounced effect (Figure 5I).

### 3.6. Effect of Acrylamide and Glycidamide on Expression of Genes Involved in MAPK Signaling Pathway

**Figure 6:**
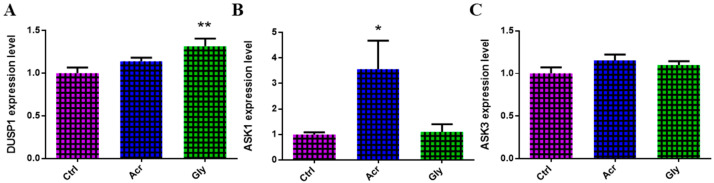
Effects of ACR and GLY on MAPK signaling pathway. (**A**–**C**) Expression levels of MAPK signaling-related genes in oocytes treated with ACR and GLY using RT-qPCR. The comparison between ACR or GLY groups versus the control was performed using Student’s *t*-test. Statistical significance is presented as * (*p* < 0.05) and ** (*p* < 0.01), with values shown as mean ± SEM.

The expression of genes involved in the MAPK signaling pathway, which plays a critical role in cellular responses to stress, was analyzed in oocytes treated with ACR and GLY using RT-qPCR. As shown in Figure 6, ACR-treated oocytes exhibited a significant upregulation in *ASK1* expression, while GLY treatment showed a significant increase in *DUSP1* expression.

### 3.7. Effect of Acrylamide and Glycidamide on the Quality of Developed Blastocysts

**Figure 7:**
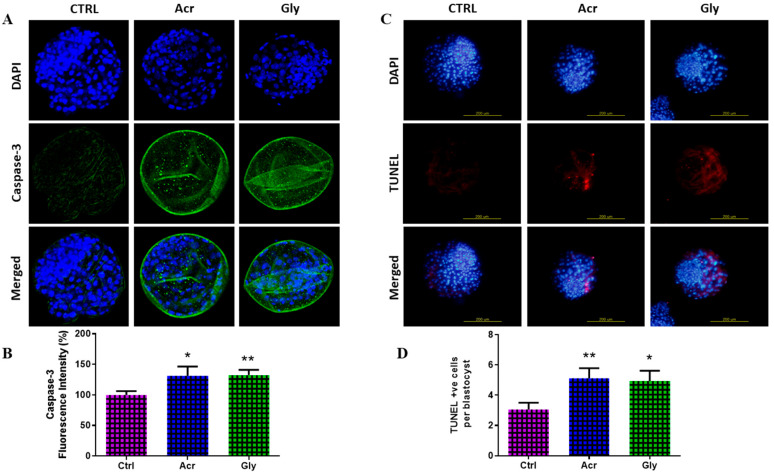
Quality of blastocysts following ACR and GLY exposure. Immunofluorescence of caspase-3 protein (**A**,**B**) and TUNEL staining (**C**,**D**) in blastocysts derived from ACR- and GLY-treated oocytes. The comparison between ACR or GLY groups versus the control was performed using Student’s *t*-test. Statistical significances are presented as * (*p* < 0.05) and ** (*p* < 0.01), with values shown as mean ± SEM. Original magnification 100× (**A**). Scale bar = 200 µm (**C**).

To investigate the effect of ACR and GLY exposure on the in vitro-developed blastocysts, the quality of embryos was assessed using caspase-3 staining and TUNEL assays to detect apoptosis. As seen in Figure 7, embryos derived from ACR- and GLY-treated oocytes showed significantly higher levels of caspase-3 and increased apoptosis compared to the control group.

## 4. Discussion

Exposure to acrylamide (ACR) and its primary metabolite, glycidamide (GLY), is nearly unavoidable due to their presence in widely consumed foods and environmental pollutants, raising significant concerns about potential toxicity to reproductive health [14,37]. Research has shown that ACR or GLY disrupt essential processes during embryo development, including meiotic progression, chromosomal integrity, and epigenetic modifications, potentially compromising fertility and embryonic viability [4,14,15]. This study aimed to assess the impact of ACR and GLY on oocyte and embryo development, with a specific focus on DNA damage, apoptosis, autophagy, and epigenetic regulation using a bovine oocyte model. The findings indicate that both ACR and GLY significantly impair oocyte developmental competence and reduce blastocyst formation rates. These results align with studies demonstrating that ACR and GLY exposure negatively affects oocyte quality through mechanisms such as oxidative stress, DNA damage, and apoptosis [4,8,14,15]. Such disruptions in cellular processes hinder oocyte maturation and progression through critical developmental stages, ultimately reducing blastocyst formation.

To assess the impact of ACR and GLY on oocyte quality, we investigated the organization of actin microfilaments during oocyte maturation, a critical factor for successful meiotic maturation and fertilization [38]. Previous research has reported that ACR disrupts actin organization in the membrane and cytoplasm of mouse oocytes [8]. Consistent with these findings, our results demonstrated similar disruptions in actin organization in oocytes treated either with ACR or GLY. These observations suggest that abnormal actin distribution may contribute to the failure of embryo cleavage observed in oocytes exposed to ACR and GLY.

Next, we examined the effects of ACR and GLY on the mRNA of certain markers involved in oocyte quality and development competency. In this regard, MARF1, a key regulator of RNA stability and genomic integrity, is essential for proper meiotic progression in oocytes [39]. In this study, oocytes treated with ACR and GLY showed a significant upregulation in MARF1 expression, suggesting that these stressors can disrupt the expression of critical genes related to oocyte quality. Additionally, GDF9, an oocyte-specific paracrine factor, plays an essential role in oocyte maturation, follicular development, and communication with surrounding granulosa cells—processes vital for successful folliculogenesis and fertility [40]. The tight regulation of GDF9 expression within the oocyte is critical, as disruptions in this pathway are associated with impaired follicle development and compromised reproductive health [41]. Our findings indicate that GLY-treated oocytes exhibited a significant decrease in GDF9 expression alongside an increase in DUSP1 expression. This alteration in gene expression could lead to compromised oocyte maturation and reduced fertility.

DNA methylation and histone modifications are two central mechanisms of epigenetic regulation, playing a pivotal role in oocyte maturation and early embryonic development [42]. Specific DNA methylation patterns established in oocytes are crucial for genomic imprinting and early embryo development [15]. In this study, we observed significant changes in the expression of key epigenetic regulators, underscoring the potential of ACR and GLY to disrupt normal developmental processes. ACR-treated oocytes exhibited upregulation of *DPPA3*, *EZH1*, and *EZH2*, while GLY-treated oocytes showed increased *SUV39H2* and *DPPA3* expression coupled with reduced *EED* levels. These alterations likely impact chromatin structure and gene expression, contributing to the impaired oocyte and embryo development [35,43]. Additionally, disruptions in histone modifications may interfere with chromosome condensation and segregation, delaying oocyte maturation [35]. *EZH1* and *EZH2* play essential roles in maintaining H3K27 methylation, which regulates gene expression during oocyte maturation and early embryogenesis [44,45]. Similarly, *DPPA3* aids DNA demethylation and chromatin organization, critical for early embryonic development [46]. Alterations in these epigenetic mechanisms may compromise chromosomal stability and gene regulation, potentially contributing to the reproductive toxicity related to ACR and GLY exposure.

This study also demonstrated a significant upregulation of apoptosis-related proteins, especially caspase-3, in oocytes exposed to both ACR and GLY. This rise in apoptosis likely contributes to the observed reduction in cleavage rates and compromised blastocyst development in the group of treated embryos. Additionally, both ACR and GLY treatments significantly increased DNA damage markers, such as *BAX*, *caspase-3*, *iNOS*, and *OGG1*, in oocytes. These findings are consistent with prior research showing ACR-induced activation of apoptotic pathways via caspase activation and mitochondrial dysfunction [47,48]. Embryo quality was further assessed using caspase-3 and TUNEL assays, which highlighted apoptosis as a key determinant of embryo quality [49]. Embryos derived from ACR- and GLY-treated oocytes exhibited higher levels of caspase-3 and apoptosis relative to controls, indicating reduced embryo quality due to increased cell death. Furthermore, ACR was shown to promote apoptosis by decreasing *Bcl-2* expression, elevating *BAX* and cleaved caspase-3 levels, and raising apoptosis rates, consistent with findings in Neuro-2a cells [50].

Autophagy is a critical process for degrading and recycling damaged organelles, maintaining intracellular balance while regulating both cell death and growth [51]. In this study, we observed increased expression of autophagy-related genes (*LC3A*, *LC3B*, *ATG7*, *Lamp1*, *Lamp2*) and elevated LC3B protein levels in oocytes treated with ACR and GLY. These findings align with previous studies indicating that ACR-induced autophagy exacerbates apoptosis in both cell and animal models [52,53]. Stressors such as oxidative damage and nutrient deprivation are known to activate autophagic processes to sustain cellular homeostasis [54,55]. Studies have shown that ACR can activate autophagy by increasing LC3 levels in SH-SY5Y cells [56] and human chondrocytes [57]. Interestingly, GLY treatment resulted in a more pronounced increase in autophagic markers compared to ACR, suggesting that GLY may induce autophagy more robustly as a compensatory mechanism to cellular stress. While autophagy may initially serve a protective function, prolonged or excessive activation can lead to autophagic cell death, potentially contributing to the impaired oocyte and embryo development observed in this study.

The MAPK signaling pathway, essential for cellular stress responses [58,59] and apoptosis [59], was disrupted by both ACR and GLY. Specifically, ACR treatment significantly elevated *ASK1* expression, while GLY treatment upregulated *DUSP1*, indicating distinct impact of these compounds on the pathway components. ASK1 activation has been associated with various pathological conditions, including apoptosis in different cell types [60]. The dysregulation of the MAPK signaling pathway not only exacerbates the toxic impact on reproductive outcomes but may also impair cellular homeostasis, contributing to cumulative stress within the oocyte. Increased p-MAPK expression further confirmed that ACR induces apoptosis, likely through oxidative stress-mediated pathways, as reported in mouse oocytes [15]. Given the MARK pathway’s role in regulating cell cycle checkpoints and stress responses, its disruption could hinder meiotic progression, ultimately reducing oocyte viability and developmental competence. These findings support prior studies suggesting that ACR triggers oxidative stress-related apoptotic death, highlighting the pathway’s critical role in safeguarding oocyte quality and fertility potential.

## 5. Conclusions

This study provides compelling evidence of the harmful effects of acrylamide and glycidamide on bovine oocyte and embryo development. The observed increases in DNA damage, apoptosis, and autophagy, along with disruptions in epigenetic regulation and signaling pathways, underscore the reproductive risks associated with exposure to these contaminants. These findings highlight the need for targeted strategies to mitigate the adverse effects of ACR and GLY to safeguard reproductive health.

## Data Availability

The original contributions presented in this study are included in the article/Supplementary Materials.

## Supplementary Materials

The following supporting information can be downloaded at https://www.mdpi.com/article/10.3390/toxics13030223/s1: Table S1: List of primers used in RT-qPCR.
