# Supplementary material for: Acrylamide and Its Metabolite Glycidamide Induce Reproductive Toxicity During In Vitro Maturation of Bovine Oocytes
Source: Toxics. 2025 Mar 19;13(3):223. doi: 10.3390/toxics13030223 (PMC11946555; doi:10.3390/toxics13030223)
Supplement: Supplementary file 1 [file toxics-13-00223-s001.zip › toxics-3474800-supplementary.pdf]

Table 1. List of primers used in RT-qPCR.

Primer name	Sequence	Accession number	Amplicon size
**Oocyte developmental markers**			
GDF9	F: GGACCCCTAAATCCAACAGA<br>R: ACAGTAACACGATCCAGGTT	NM_174681.2	123
BMP15	F: TCCAGAACCTTGTCAATGAG<br>R: GGGCAATCATACCCTCATAC	NM_001031752.1	141
MARF1	F: GCAGAGCACCAGGACAATCA<br>R: GAAATAGCCCGCAGAGGAAG	XM_015104092.2	262
**DNA and Histone methylation**			
DNMT1A	F: ACGAATGGTGGATTGCTGGT<br>R: CAGGTCTTCGTAGGTGGAGTC	XM_027547790.1	197
DNMT3A	F: AGACGGCAAGTTCTCAGTGG<br>R: ACCTGCAGGACCTCGTAGAT	NM_001206502.2	129
DNMT3B	F: TCTCCAATGTGAACCCTGCC<br>R: CATCCAGAAGAAAGGCCGGT	XM_027558803.1	125
G9A	F: GATTTCCACGCATCGCCTTC<br>R: GCACTTCTCAGAGCCACACT	XM_024983572.1	131
SETDB1	F: TCTATCGAGGCTCTACGCGA<br>R: TGGACGTGTTCTGAGCTGTC	NM_001191388.3	101
SUV39H2	F: GTGTCCAACGTGACAGCAAC<br>R: AGTGCTGCATCCCTGAAGTC	XM_005214231.4	100
EZH1	F: ATCTTCCGGGTAGTGGGGTG<br>R: ATGGAACCTGGGAGCTCCTC	XM_015458835.2	144
EZH2	F: GCAACGACAAGGACGAGGAG<br>R: AGTAGGTGCCGATGAGGACC	XM_024990587.1	168
SUZ12	F: GATATTCATCGCCAGCCCGG<br>R: GGCGGTTGTGTCCACTACTG	NM_001205587.3	178
EED	F: TAGCAACCCGGACCTCTCTG<br>R: TCCTTCCAGGTGCATTTGGC	XM_015461113.2	113
**Inflammation/apoptosis and cell cycle Regulators**			
BAX	F: CACCAAGAAGCTGAGCGAGTGT<br>R: TCGGAAAAAGACCTCTCGGGGA	NM_173894	118
BCL2	F: TGGATGACCGAGTACCTGAA<br>R: CAGCCAGGAGAAATCAAACA	NM_001166486.1	120
NF-kB	F: TGGCGGAATTACCTTCCATAC<br>R: CATCACTCTTGCCACAACTTTC	DQ464067	110
TXN	F: TCGGATCCGTGTCCATCGAT<br>R: CACGTGGCTGAGAAGTCGAC	AF104105.1	151

Caspase-3	F: CCCAAGTGTGACCACTGAAC<br>R: CCATTAGGCCACACTCACTG	NM_001077840.1	169
Caspase-9	F: CGCCACCATCTTCTCCCTG<br>R: CCAACGTCTCCTTCTCCTCC	NM_001077111.1	83
iNOS2	F: CGAGCTTCTACCTCAAGCTATC<br>R: CTGGCCAGATGTTCCTCTATTT	DQ676956.1	84
CYTC	F: CCAGGTAGCCAAGGATGTGT<br>R: CTTTCGGCTCTTGAGGACTG	XR_003038028.1	163
COX2	F: CTTAAACAAGAGCATCCAGAATGG<br>R:<br>GCTGTACGTAGTCTTCAATCACAATCT	NM_174445.2	106
P21	F: GCAAATATGGGTCTGGGAGA<br>R: AAATAGTCCAGGCCAGGATG	NM_001098958.2	112
P27	F: TGTCAAACGTGCGAGTGTCTA<br>R: CTCTGCAGTGCTTCTCCAAGT	XM_019961532.1	150
**DNA repair and damage response**			
P53	F: CTATGAGATGTTCCGAGAGC<br>R: CTCTCTCTTGAGCATTGGTT	NM_174201.2	153
OGG1	F: CAACTCCAGCAAGCTCAGGA<br>R: GAGACTGGATGGGGGAGAGT	XM_010817459.3	140
PP6	F: CCGTCGGGGCTTCTTACATC<br>R: CGCTTCAAGTCGTTCTCCGG	NM_001130750.1	101
**Autophagy markers**			
BECN1	F: AGTTGAGAAAGGCGAGACAC<br>R: GATGGAATAGGAACCACCAC	NM_001033627.2	100
LC3A	F: CATGAGCGAGTTGGTCAAAA<br>R: GGGAGGCGTAGACCATGTAG	XM_027558753.1	170
LC3B	F: TTATCCGAGAGCAGCATCC<br>R: AGGCTTGATTAGCATTGAGC	XM_027513856.1	171
ATG5	F: CCACTGCCGTCATTAAACCT<br>R: TTCCACTCCCTCGAGCTAAA	XM_024996700.1	212
ATG7	F: ATGGCCTTTGAGGAACCTTT<br>R: ATGCCTCCCTTCTGGTTCTT	XM_010817935.3	210
BNIP3	F: GAAGGAATGCCGACACTAGG<br>R: CAAAGCCAGCAGACACTCAG	XM_027528566.1	138
Lamp1	F: GTGAAGAATGGCAACGGAC<br>R: GCATCAGCTGGACCTCGTAA	XM_027558031.1	250
Lamp2	F: AAGAGCAGACCGTTTCCGTG	XM_027535042.1	110

	R: CGAACACTCTTGGGCAGTAG		
**MAPK signal transduction pathway**			
DUSP1	F: CGATCTTCACAACCCACCGG<br>R: GCCCCCTTCCCTGGAGTTAT	NM_001046452.2	70
ASK1	F: ACGGGATCAGTCAGGTCCAG<br>R: AAATGCGGCCTCAGTTCTGG	NM_001144081.2	169
ASK3	F: AGGAGCAGAATCAGGTGGCT<br>R: GTGGATGCCATCACCCTGTG	XM_010822151.3	148
**Reference Gene**			
GADPH	F: CCCAGAATATCATCCCTGCT<br>R: CTGCTTCACCACCTTCTTGA	NM_001034034.2	185

ABV: Growth Differentiation Factor 9 (*GDF9*), Bone Morphogenetic Protein 15 (*BMP15*), Meiosis Arrest Female 1 (*MARF1*), epigenetic modifiers: DNA Methyltransferase 1 Alpha (*DNMT1A*), DNA Methyltransferase 3 Alpha (*DNMT3A*), DNA Methyltransferase 3 Beta (*DNMT3β*), Euchromatic Histone-Lysine N-Methyltransferase 2 (*G9A*), SET Domain Bifurcated Histone Lysine Methyltransferase 1 (*SETDB1*), Suppressor of Variegation 3-9 Homolog 2 (*SUV39H2*), Developmental Pluripotency Associated 3 (*DPPA3*), Enhancer of Zeste Homolog 1 (*EZH1*), Enhancer of Zeste Homolog 2 (*EZH2*), Suppressor of Zeste 12 Homolog (*SUZ12*), Embryonic Ectoderm Development (*EED*), BCL2 Associated X (*BAX*), inflammation/apoptosis and cell cycle regulators: B-Cell Lymphoma 2 (*BCL2*), Nuclear Factor Kappa B (*NFkB*), Thioredoxin (*TXN*), Inducible Nitric Oxide Synthase (*iNOS*), Cytochrome C (*CYTC*), Cyclooxygenase 2 (*COX2*), Cyclin-Dependent Kinase Inhibitor 1 (*P21*), Cyclin-Dependent Kinase Inhibitor 1B (*P27*), DNA repair and damage response: 8-Oxoguanine DNA Glycosylase (*OGG1*), Tumor Protein P53 (*P53*), Protein Phosphatase 6 (*PP6*), autophagy-related genes: Beclin-1 (*BECN1*), Microtubule-Associated Proteins 1A/1B Light Chain 3A (*LC3A*), Microtubule-Associated Proteins 1A/1B Light Chain 3B (*LC3B*), Autophagy Related 5 (*ATG5*), Autophagy Related 7 (*ATG7*), BCL2 Interacting Protein 3 (*BNIP3*), Lysosomal Associated Membrane Protein 1 (*Lamp1*), Lysosomal Associated Membrane Protein 2 (*Lamp2*), signal transduction pathways: Dual Specificity Phosphatase 1 (*DUSP1*), Apoptosis Signal-Regulating Kinase 1 (*ASK1*), Apoptosis Signal-Regulating Kinase 3 (*ASK3*), glyceraldehyde-3-phosphate dehydrogenase (GAPDH).
